# Biocontrol of multi-drug resistant pathogenic bacteria in drainage water by locally isolated bacteriophage

**DOI:** 10.1186/s12866-023-02847-4

**Published:** 2023-04-26

**Authors:** Rabab M. Soliman, Badawi A. Othman, Sahar A. Shoman, Mohamed I. Azzam, Marwa M. Gado

**Affiliations:** 1 Department of Microbiology, Faculty of Science, Ain Shams University, Abbassia, Cairo, 11566 Egypt; 2 Department of Agricultural Microbiology, Faculty of Agriculture, Ain Shams University, Hadayek Shubra 11241, Qalibia, Egypt; 3 Department of Microbiology, Central Laboratory for Environmental Quality Monitoring (CLEQM), National Water Research Center (NWRC), El-Kanater El-Khairia 13621/6, Qalibia, Egypt

**Keywords:** Drainage water, *E. coli*, *P. aeruginosa*, MAR index, Phage cocktail

## Abstract

In areas with limited water resources, the reuse of treated drainage water for non-potable purposes is increasingly recognised as a valuable and sustainable water resource. Numerous pathogenic bacteria found in drainage water have a detrimental impact on public health. The emergence of antibiotic-resistant bacteria and the current worldwide delay in the production of new antibiotics may make the issue of this microbial water pollution even more challenging. This challenge aided the resumption of phage treatment to address this alarming issue. In this study, strains of *Escherichia coli* and *Pseudomonas aeruginosa* and their phages were isolated from drainage and surface water from Bahr El-Baqar and El-Manzala Lake in Damietta governorate, Egypt. Bacterial strains were identified by microscopical and biochemical examinations which were confirmed by 16 S rDNA sequencing. The susceptibility of these bacteria to several antibiotics revealed that most of the isolates had multiple antibiotic resistances (MAR). The calculated MAR index values (> 0.25) categorized study sites as potentially hazardous to health. Lytic bacteriophages against these multidrug-resistant strains of *E. coli* and *P. aeruginosa* were isolated and characterized. The isolated phages were found to be pH and heat stable and were all members of the *Caudovirales* order as recognized by the electron microscope. They infect 88.9% of *E. coli* strains and 100% of *P. aeruginosa* strains examined. Under laboratory conditions, the use of a phage cocktail resulted in a considerable reduction in bacterial growth. The removal efficiency (%) for *E. coli* and *P. aeruginosa* colonies increased with time and maximized at 24 h revealing a nearly 100% reduction after incubation with the phage mixture. The study candidates new phages for detecting and controlling other bacterial pathogens of public health concern to limit water pollution and maintain adequate hygiene.

**Supplementary Information:**

The online version contains supplementary material available at 10.1186/s12866-023-02847-4.

## Introduction

In Egypt, agriculture uses the most readily available water, accounting for more than 85% of the total demand; hence, drainage water is recycled to close the gap between supply and demand [1]. Bahr El-Baqar drain is considered one of the most polluted drains in Egypt. That drain pumps 2.3 BCM of water annually into Lake Manzala. It is passing through the heavily populated governorates of Qalubeya, Sharkia, Ismailia, and Port Said. Particulate, nutrient, heavy metal, hydrocarbon, and hazardous chemical residues, such as herbicide and pesticide residues, make up the wastewater in the drain [2]. Although Bahr El-Baqr is the largest drain in the Eastern Delta, its high degree of pollution prevented it from being connected to the El-Salam Canal, which transports mixed drainage and fresh water to Sinai. Large volumes of water that could have been used for irrigation are lost as a result of this problem [3].

Numerous bacteria, including gastrointestinal pathogens, are present in drainage water *(E. coli, Proteus sp, Salmonella sp, Shigella sp, Citrobacter sp)* which causes diarrhoea in both humans and animals when it enters the gastrointestinal tract. Diarrheal illness was considered the third-leading cause of child death worldwide in 2017, which killed more than 500,000 children under the age of five [4]. These intestinal infections can spread if industrial and livestock wastes are not properly controlled.

The rise of antibiotic-resistant bacteria may make the issue of microbiological water contamination even more challenging [5]. The issues with multidrug-resistant Gram-negative bacteria (MDRGNB) are particularly alarming. These bacteria include *Klebsiella sp, Enterobacter sp, E. coli, P. aeruginosa* and *Acinetobacter sp.* These problems are primarily caused by the overuse and abuse of antibiotics [6].

*E. coli* is an indicator bacterium that gives clear proof of recent faecal contamination. Because of the various pathogenicity mechanisms and illnesses that it can cause, *E. coli* is the most notable example of a gram-negative bacterium associated with a variety of diseases [7]. *P. aeruginosa* is one of the most often discovered multidrug-resistant organisms in the world [8].

Phage treatment has been researched due to the global spread of multidrug-resistant (MDR) bacterial infections [9]. These bacteriophages, in contrast to conventional broad-spectrum antibiotics, target particular bacteria without damaging others. In the lytic (or virulent) lifestyle, the host cell’s replication and eventual destruction are facilitated by hijacking the machinery. This results in the production of progeny while also killing the host [8, 10]. The enormous variety and abundance of phages found in nature make it a simple source which is chosen for a range of applications, such as decontamination, infection control, detection, diagnosis, anti-bacterial therapy and phage typing. Bacteriophages can be used as bacterial pathogen indicators and as biocontrol agents in wastewater treatment [11].

In response to previous challenges, the present study was conducted to evaluate the efficacy of a newly isolated phage cocktail for detecting and reduction of some gram-negative bacteria in water, being the best control model for water-born opportunistic pathogens of public health concern.

## Materials and methods

### Collection of water samples

The study was carried out between May 2018 and June 2019. An overall total of 75 water samples were collected from the Bahr El-Baqr drain outlet, pump station, sedimentation basin, drying basin, surface flow basin, subsurface flow basin, and exits from Lake Manzala.

All analyses were done according to the Water and Wastewater Examination Standards of the American public health association [12]. Water samples were taken from the subsurface layer at a depth of 50 cm and placed in sterile, clean polyethene plastic bottles. All samples for physical, chemical, or bacteriological investigation were kept in ice boxes and submitted right away to the lab for examination. All analyses were carried out in the Central Laboratory for Environmental Quality Monitoring (CLEQM) National Water Research Center (NWRC), Cairo, Egypt, Virology Lab., Agric., Microbiology Dept. Fac. of Agriculture, Ain shams University, Shobra El-Kheima, Qalubia Governorate, Egypt and bacteriology Lab., Sci., Microbiology Dept. Fac. of Science, Ain shams University, Alabbasia, Cairo Governorate, Egypt.

### Isolation and identification of specific bacteria

*E. coli* and *P. aeruginosa* were the most common pathogens found in wastewater. Water samples were quickly filtered using a 0.45 µ m membrane, the membrane was plated on m-TEC agar medium for *E. coli* and M-PA-C agar medium for *P. aeruginosa*. Typical colonies were sub-cultured for purification and confirmation on Eosin Methylene Blue agar plates for *E. coli* and Cetrimide agar for *P. aeruginosa*. Gram staining, colony morphology, and other traditional biochemical methods were used to identify bacterial isolates [13].

### Molecular identification of bacterial isolates

Genomic DNA was extracted using Bacterial Genomic DNA Isolation Kit RKT09 (Chromous Biotech Pvt. Ltd., Bangalore, India) and visualized on 0.8% (w/v) agarose gel. Gene amplification was carried out using a Thermal cycler (ABI 2720) in 100 µl reaction volume containing 2.5 mM of dNTP, 10x PCR buffer, 3U of Taq DNA polymerase, 10 ng template DNA, and 400 ng of primer (F) 5’-GGGGGATCTTCGGACCTCA-3’, and primer (R) 5’-TCCTTAGAGTGCCCACCCG-3’ which were designed for aquatic gram-negative bacteria [14, 15]. The sequencing was performed according to the manufacturer’s protocol using Big Dye Terminator Cycle Sequencing Kit (V. 3.1, Applied Bio-system) and analyzed in an Applied Bio-system analyzer. The sequences of 16 S-rDNA for these strains were finally submitted to the NCBI GenBank database, USA, and compared to other available sequences using an automated alignment tool blast program and assigned their accession numbers [16].

### Pathogenicity test for bacterial isolates

This test was carried out by using the Congo Red Agar (CRA) method [17]. On Congo red agar medium, single colonies from each isolate of a 24-hr old pure bacterial culture were streaked. For easier result annotation, the cultures were kept at room temperature for 48 h after 24 h of incubation at 35 ^o^C. Colonies that appeared red were noted as positive results and considered to be pathogen isolates (virulent). Colonies that were not red were considered to be non-pathogen isolates and were recorded as negative results (avirulent).

### Antibiotic sensitivity test for bacterial isolates

Eleven commercially manufactured antibiotic discs from Oxoid-UK were selected for this study to examine their effectiveness against bacteria isolated from water samples. These drugs belonged to eleven classes of antibiotics: Penicillin, Cephalosporins, Glycopeptides, Aminoglycosides, Tetracyclines, Macrolides, Lincosamides, Quinolones, Sulfa drugs, Nitrofurans and Chloramphenicol. The standard agar disk diffusion method was performed [18]. According to protocols defined for the assessment of antibiotic compounds as directed by the National Committee for Clinical Laboratory Standards, the diameters of the inhibition zones surrounding the antibiotic discs were measured to the nearest whole millimetre and reported [19].

### Multiple antibiotics resistance indexing

The Multiple antibiotic resistance (MAR) index was calculated according to the following formula:

MAR = X/nY.

Where:

X: the number of resistances determined among population “Y”.

n: the number of tested antibiotics.

*Values of MAR higher than 0.25 pose a high-risk source of contamination [20, 21].

## Detection of bacteriophages

### Isolation and enrichment of *E. coli* and *P. aeruginosa* phages

The phages specific for isolated and identified bacterial strains of *E. coli* and *P. aeruginosa* collected from water samples were detected [22]. Five ml of the obtained water samples were added to Erlenmeyer flasks (250 ml), each of which contained 50 ml of nutrient broth. Mixtures of each strain (1ml) were added, and the flasks were incubated at 37 °C for 72 h, with shaking at 150 rpm using shaking incubator. After incubation, the liquid cultures were centrifuged at 6000 rpm for 15 min to remove the cell culture debris. Chloroform was added to the supernatant (1:10 v/v), followed by a vigorously shaking for 3–5 min, then the flasks were allowed to be settled for 30 min to remove any contaminating bacteria.

### Assaying of *E. coli* and *P. aeruginosa* phages

Bacteriophages were detected both qualitatively and quantitatively using the spot test and over layer agar techniques (Plaque assay technique) [22].

### Chemical purification and concentration of isolated phages

Phage purification and concentration were carried out using a two-phase liquid system with dextran sulphate and polyethylene glycol [23]. The mixture centrifuged at 2000 rpm for l0min. Then, the supernatant was collected and centrifuged at 16,000 rpm for 2 h at 4 °C. In order to conduct the analysis, the phage pellets were resuspended in 1ml of saline solution.

### Characterization of *E. coli* and *P. aeruginosa* phages

#### Electron microscopy examination

One drop of each isolated phage suspension (10^12^ pfu/ml) was placed on a 200 mesh carbon-coated copper grid and allowed to absorb for approximately 20 min, the excess liquid was removed with filter paper dick. The grids were negatively stained with 2% uranyl acetate (pH 4.5) for 90 s and left for drying and then examined using a JOEL-JEM- 1010 electron microscope operated at 80 KV (Electron microscope unit, Regional Center for Mycology and Biotechnology, Al-Azhar Univ., Cairo) [24].

#### Host range pattern

Agar double-layer plates were used for host range assay. *P. aeruginosa, P. aeruginosa ATCC 27,853 and E. coli, E. coli* ATCC 25,922 and *E. coli* ATCC 10,536 were used as different host in individual plates. ATCC strains were obtained from the Egyptian Microbial Culture Collection (EMCC) at Cairo Microbial Resource Center. Plates were prepared by pouring a base layer of 45 ml of nutrient agar medium with 1.5% agar in Petri dishes 10 cm in diameter. The basal layer was allowed to solidify. A mixture of 3 ml melted semi-solid agar and 1 ml of the bacterial host were poured into each plate, 24 h incubation of the used strains. The surface of every plate was spotted with 5 µL of every single phage isolate. After incubation for 24 h at 37 °C, plates were examined for lysis of bacteria at the sites where the drops had been applied and the obtained data were recorded.

### Physical properties of virions

#### Thermal stability

Two ml of each phage suspension were incubated at 40 ºC, 50 ºC, 60 ºC, 70 ºC, 80 ºC, 90 ºC and 100 ºC for 10 min with sudden cooling. Phage infectivity was determined by plaque assay technique [25].

#### PH stability

One ml of each phage suspension was transferred into test tubes containing 9 ml of nutrient broth. The pH of the total suspension was adjusted at various values from 4 to 12. The pH values of the nutrient broth were adjusted using 0.1 M HCl or NaOH. After an hour of incubation at 37 ºC, the infectivity of phages was determined by carrying out a double-layer technique [26].

### Challenging with phage cocktail

Investigation of the effects of identified phages on decreasing the viable count of the *E. coli* and *P. aeruginosa* bacteria was detected in sewage water samples.

Water samples collected from the Bahr El-Baqar drain were divided into 3 parts (2 L for each part). Two L was used as control, 2 L of wastewater was treated with a cocktail of coliphage, and 2 L was treated with a cocktail of *E. coli* or *Pseudomonas* phages. The phage cocktail was prepared by mixing equal volumes of isolated phages (10 ml of each phage) specific to bacterial hosts (*E. coli* and *P. aeruginosa*) at a concentration of 1012 PFU/ml as determined by plaque assay.

Water samples collected from the Bahr El-Baqar drain were inoculated with a phages cocktail (1 L/1 ml) and incubated at (37 ◦C and 150 rpm). Both *E. coli* and *P. aeruginosa* colonies were counted at different time points (2, 4, 6, 8, 10, 12, 24 h) incubation. After each incubation period, each 100 ml of treated water was filtered. Plates with specific media incubated with the membrane for 24 h at 44.5 ± 0.2◦C for *E. coli* and 72 h at 41.5 ± 0.5 oC for *P. aeruginosa*. Finally, bacterial colonies were counted and the average results were recorded in (cfu/100ml).

The efficiency of bacteriophages was detected by counting bacterial colonies before and after treatment [7]. The removal efficiency (%) for *E. coli and P. aeruginosa was* calculated as the following equation:

Removal efficiency = (count before treatment - count after treatment)***/*count before treatment x100**.

## Results

### Isolation and identification of specific bacterial isolates

E. coli. *   E. coli* isolates gave red to magenta colonies on the membrane filter after incubation on a modified mTEC agar medium. It was isolated and confirmed by streaking on MacConkey agar medium giving pink growth and on Eosin Methylene Blue (EMB) agar medium showing purple growth with a green metallic sheen.

- *P. aeruginosa* colonies showed a flat appearance with light outer rims, brownish to greenish black centres and 0.8 to 2.2 mm in diameter on membrane filter after incubation on M-PA-C agar medium. By streaking on cetrimide agar plates, it appeared as blue-green colonies on plates.

#### Molecular identification of bacterial isolates

The PCR-amplified products were separated into two unique fragments with varying molecular weights: R1 (818 bp) and R2 (1119 bp) Using 2% agarose gel electrophoresis. The nucleotide sequences of the two Egyptian strains were compared with strains from other regions using multiple sequence alignment (MSA). The NCBI GenBank database in the USA received nucleotide sequence submissions, which were given the accession numbers MK064165.1 and MK071734.1, respectively. Relying on MSA analysis, a phylogenetic tree was built to demonstrate the genetic relationships between the GenBank recorded strains of *P. aeruginosa* strain R2 and *E. coli* strain R1, as well as other recorded strains [13].

#### In vitro virulence ability

The ability to differentiate between pathogenic and non-pathogenic bacteria is based on the ability to bind CR dye in Congo red media, which is an essential phenotypic marker. Isolates that bind to CR dye in Congo red media generate red colonies, which are referred to be virulent isolates. When isolates lose their ability to attach to the CR, they lose their virulence and grow into white, yellow, or any colour other than red colonies. As shown in Table 1, Congo red was positive for 96% of *P. aeruginosa* isolates followed by *E. coli* (83%).

**Table 1:** Pathogenicity test for bacterial isolates from water resources

Bacterial isolate	Total isolates	No. of pathogenic isolates	percentage	No. of non-pathogenic isolates	percentage
***E. coli***	**90**	75	83%	15	17%
***P. aeruginosa***	**25**	24	96%	1	4%
**Total**	**115**	99	86%	16	14%

### Antibiotic sensitivity test for bacterial isolates

Bacterial isolates were classified as sensitive (S), intermediate (I) or resistant (R) to each antibiotic type according to NCCLS/CCLS [19], as presented in Table 2. The Most of *E. coli* isolate*s* collected from different sites were resistant to cephalothin and Clindamycin (94.4%) followed by Erythromycin (78.9%), Amoxycillin/Clavulanate and (72%), vancomycin (71%) and sensitive to Nitrofurantoin, chloramphenicol, Ofloxacin, sulfa/trimethoprim, Tetracycline and Kanamycin. All *P. aeruginosa* isolates were resistant to cephalothin, Kanamycin, Erythromycin, Clindamycin, sulfa/trimethoprim, Nitrofurantoin, and chloramphenicol. This was followed by vancomycin (88%), Tetracycline and Amoxycillin/Clavulanate (72%) while they were sensitive to Ofloxacin.

**Table 2:** Resistance pattern of *E. coli and P. aeruginosa* strains against individual antibiotics

Antibiotic	*E. coli*			*P. aeruginosa*		
	S (%)	I (%)	R (%)	S (%)	I (%)	R (%)
**Amoxycillin/Clavulanate**	13(14.5%)	12(13.5%)	65(72%)	7(28%)	0	18(72%)
**Cephalothin**	5(5.6%)	0	85(94.4%)	0	0	25(100%)
**Vancomycin**	26 (29%)	0	64(71%)	3(12%)	0	22(88%)
**Kanamycin**	55(61%)	0	35(39%)	0	0	25(100%)
**Tetracycline**	51(56.7%)	13(14.3%)	26(29%)	7(28%)	0	18(72%)
**Erythromycin**	13(14.4%)	6(6.7%)	71(78.9%)	0	0	25(100%)
**Clindamycin**	0(0%)	5(5.6%)	85(94.4%)	0	0	25(100%)
**Ofloxacin**	52(57.8%)	26(28.8%)	12(13.4%)	15(60%)	3(12%)	7(28%)
**Trimethoprim/Sulfameth-oxazole**	51(56.7%)	0	39(43.3%)	0	0	25(100%)
**Nitrofurantoin**	50(55.6%)	0	40(44.4%)	0	0	25(100%)
**Chloramphenicol**	50(55.6%)	0	40(44.4%)	0	0	25(100%)

### Multiple antibiotics resistance (MAR) index

Susceptibility of bacteria to different antibiotics (11 items) showed multiple antibiotics resistance (MAR) for the most of isolates. Calculations of MAR for individual bacterial species revealed that the most MAR values were recorded by *P. aeruginosa* (0.90) and then *E. coli* (0.45). Calculated MAR index values (above 0.25) classified the area of study as a potentially health-risk environment.

### Detection of E. coli and P. aeruginosa phages

Bacteriophages specific for *E. coli* were isolated from Bahr El-Baqar Drain outlet (1), Manzala wetland: water pumping station (2), sedimentation basins (3), drying basins (4), surface flow basins (5), subsurface flow basins (6) and Manzala Lake (7) water. The results of the spot test technique showed bacterial lysis in the form of the spot area within the *E. coli* lawn. Similar results were obtained also for *P. aeruginosa* phages.

## Characterization of the isolated phages

### Morphological characteristics of virions

**Fig. 1:**
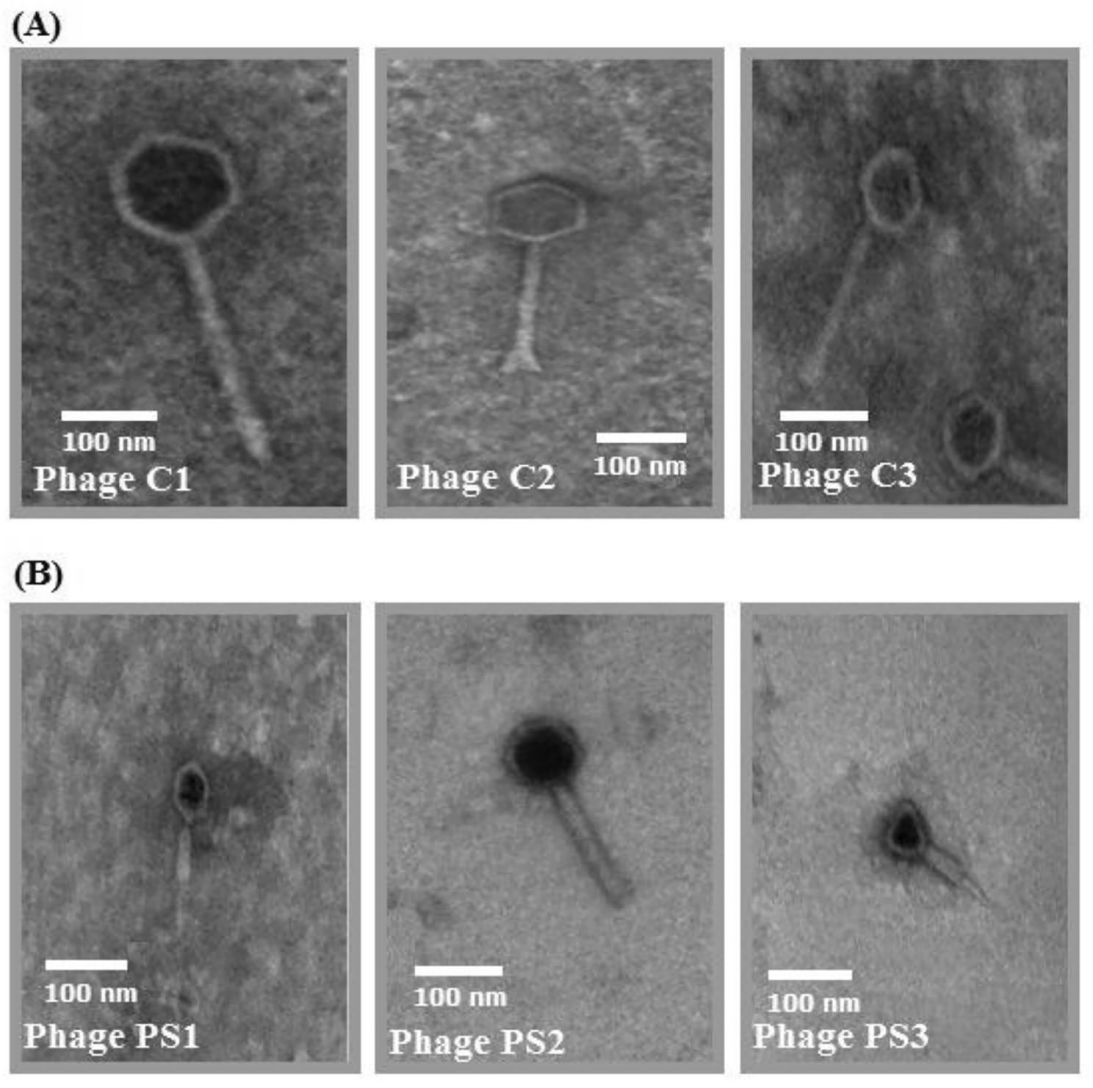
Electron micrographs of isolated phages negatively stained with uranyl acetate. (**A**) Coliphage isolates C1, C2 and C3, respectively. (**B**) *P. aeruginosa* phage isolates Ps1, Ps2 and Ps3, respectively

Electron microscopy of the isolated *E. coli* and *P. aeruginosa* phages particles revealed the three isolated coliphages and the three *P. aeruginosa* phages had an isometric head and tail. The bacteriophage resembles those of the Myoviridae and Siphoviridae families according to the International Committee on Taxonomy of viruses **(ICTV)** as shown in Table 3 and illustrated by Fig. 1.

**Table 3:** Morphological properties of six phage isolates specific for *E. coli* and *P. aeruginosa* as identified by TEM.

Phage Isolates	Family	Head capsid (nm)	Tail (nm)	
		Diameter	Length	width
C1	Siphoviridae	116.7	291.7	25
C2	Siphoviridae	120	280	30
C3	Siphoviridae	113.6	236.4	22.7
Ps1	Myoviridae	36	38.9	9
Ps2	Siphoviridae	104.2	179.2	25
Ps3	Myoviridae	50	60	23.3

### Host range of the isolated phages

Lysis of different strains of both *E. coli* and *P. aeruginosa* by the phage isolates C1, C2, C3 and Ps1, Ps2 and Ps3, respectively were examined by the spot test. Data revealed that phages C1, C2 and C3 lysed *E. coli* R1 and *E. coli***ATCC 25,922** strains while failed to lyse *E. coli***ATCC 10,536**. On the other hand, the phages Ps1, Ps2 and Ps3 lysed *P. aeruginosa* R2 *and P. aeruginosa***ATCC 27853***strains* as shown in Table 4.

**Table 4:** Host range pattern of the isolated phages to different strains of *E. coli* and *P. aeruginosa*

Phage	*E. coli* R1	*E. coli* ATCC 25,922	*E. coli* ATCC 10,536	*P.aeruginosa R2*	*P.aeruginosa ATCC* 27,853
C1	**+**	**+**	**-**	**-**	**-**
C2	**+**	**+**	**-**	**-**	**-**
C3	**+**	**+**	**-**	**-**	**-**
P1	**-**	**-**	**-**	**+**	**+**
P2	**-**	**-**	**-**	**+**	**+**
P3	**-**	**-**	**-**	**+**	**+**

**+ = lysis - = resistance (No lysis)**

### Thermal inactivation point (TIP) of the isolated phages

**Fig. 2:**
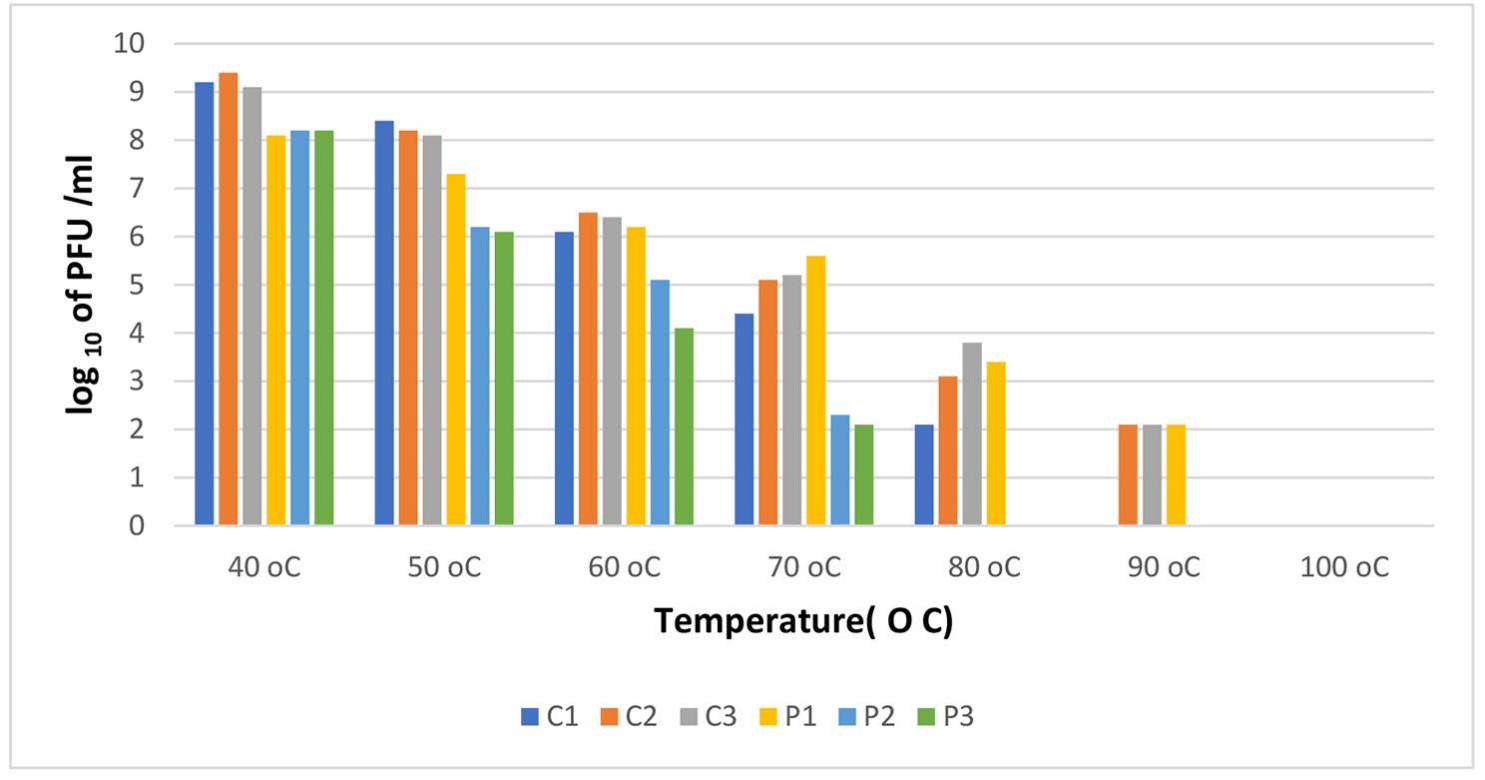
Thermal inactivation point of the isolated phages specific for both *E. coli and P. aeruginosa*

As represented in Fig. 2, E. *coli* phages as well as *P. aeruginosa* phages were affected by the heat and the rate of inhibition was increased portionaly with the heat degree. The TIP of *E. coli* C1phage was 90 ^o^C, while it was 100 ^o^C for C2 and C3. The TIP of the Ps1 phage was 90 ^o^C, and it was 80 ^o^C for both Ps2 and Ps3 phages.

### Stability of the isolated phages to acidic and alkaline conditions

**Fig. 3:**
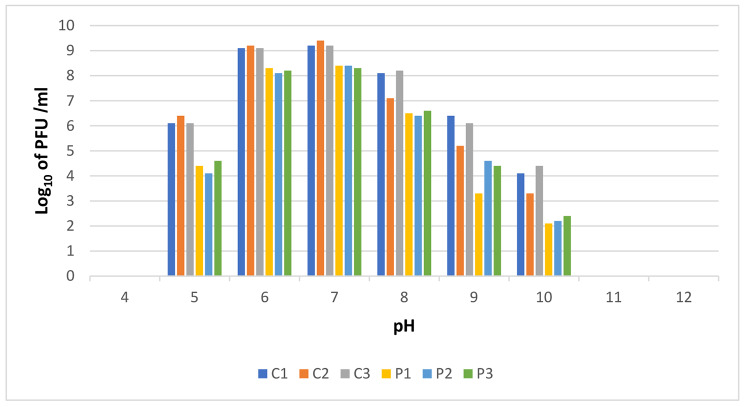
Effect of different pH values on phages infectivity

The effect of pH values of the medium on the phage activity was examined by the exposure of phage particles to pH from 4 to 12 for 1 h at 37^o^C. Results in Fig. 3 showed full inhibition was found in all phages when exposed to pH 4, 11 and 12. The optimum pH value for the phage activation was pH 7 for all phages. The phage particles tolerated the alkali media more than the acidic media.

### Effect of *E.coli and P.aeruginosa* phages on bacterial load in their aquatic environment

**Fig. 4:**
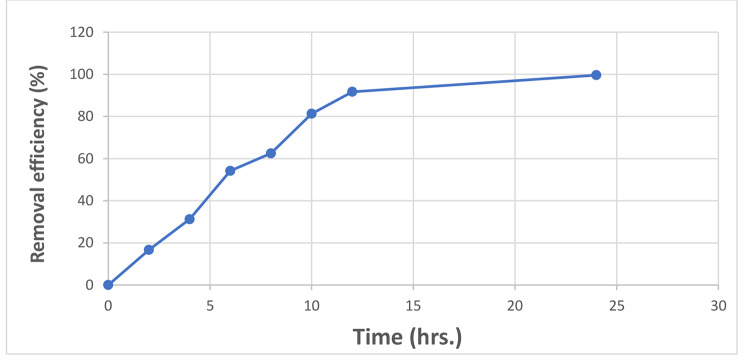
Removal efficiency (%) for *E. coli* strains was gradually increasing in a time- dependent manner

**Fig. 5:**
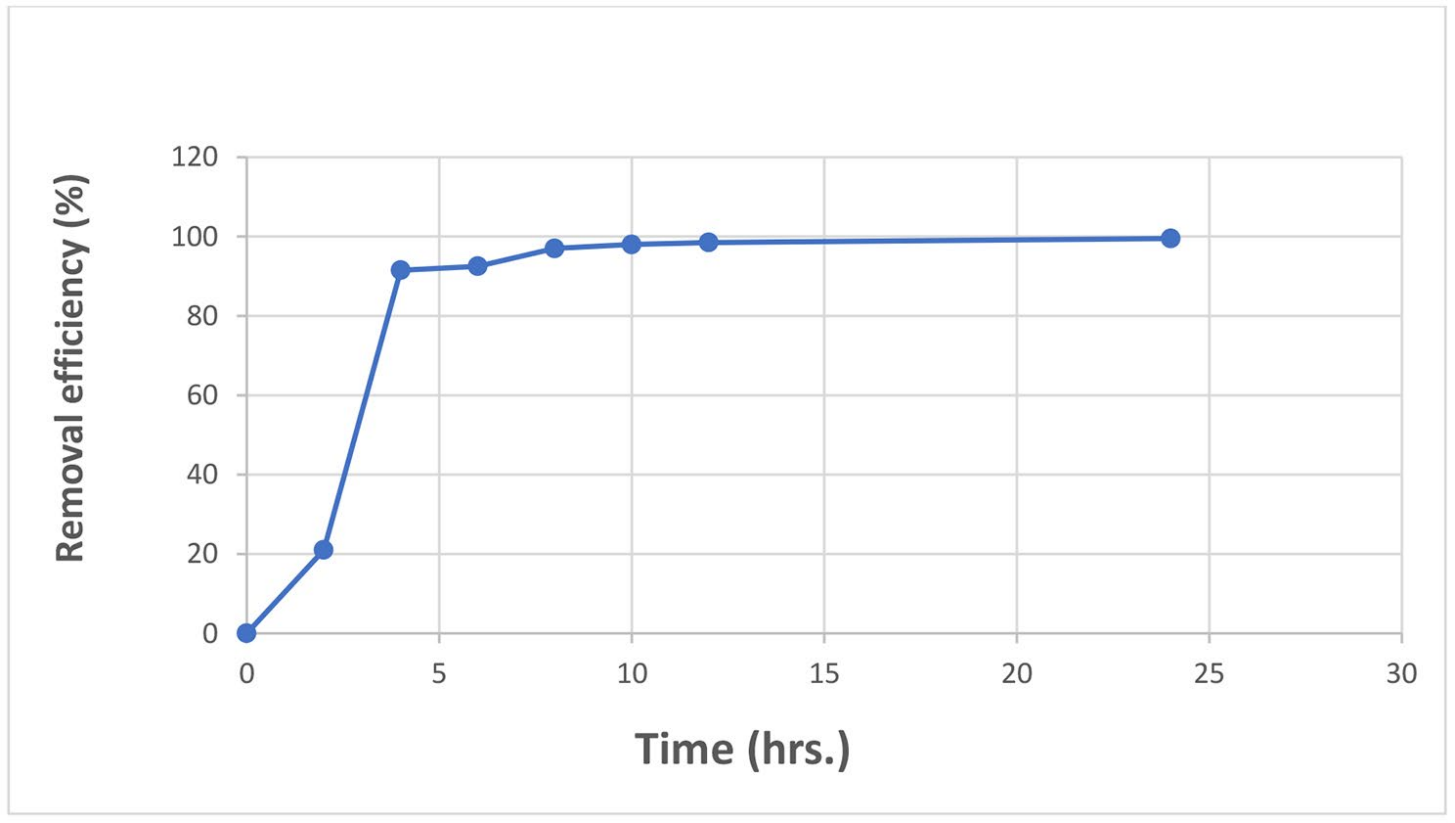
Removal efficiency (%) for *P. aeruginosa* strains was gradually increasing in a time-dependent manner

Application of *E. coli and P. aeruginosa* bacteriophages for reduction of both bacterial host numbers in drainage water of Bahr El-Baqar was carried out. Data presented in Figs. (4& 5) showed bacteriophages specific for *E. coli* reduced the total numbers of E. *coli* that naturally occurred in the drainage water with the rate of 16.7, 31.2, 54.2, 62.5, 81.3, 91.7 and 99.6% after 2 h, 4 h, 6 h, 8 h, 10 h,12 and 24 h, respectively from the addition of the bacteriophages to the crude drain water. The addition of the phages specific for *P. aeruginosa* to the crude drain water with the rate of 0.001 ml to 1000 ml reduced the total number naturally occurred in the drainage water with the rate of 21.0, 91.5, 92.5, 97.0, 98.0, 98.5 and 99.5% after 2 h, 4 h, 6 h, 8 h, 10 h,12 and 24 h, respectively.

Removal efficiency (%) for *E. coli* and *P. aeruginosa* strains was gradually increased in a time-dependent manner and peaked at 24 h post incubation with a phages cocktail. In wastewater samples, *E. coli* and *P. aeruginosa* strains revealed at least 99% reduction post 24 h of treatment with the mixture of phages. Data revealed that bacteriophages can be used to reduce bacterial load.

## Discussion

Waterborne pathogenic bacteria, such as *E. coli* and *P. aeruginosa* in water streams continue to be one of the most serious public health concerns, owing to their high expense of removal via traditional techniques in sewage treatment plants, as well as their morbidity, mortality, and destruction of nature [27]. Current wastewater treatment activities have shifted away from physical and chemical technologies, in search of new ways to efficiently eradicate those deadly pathogenic microbes. Selectable phages have shown to be useful biological control agents because of their great specificity, and efficacy in eliminating their many targeting bacterial hosts [28, 29]. Lytic phages have proven to be beneficial in sewage treatment plants, especially in activated sludge processes. By eliminating the bacteria that cause foam, they could be employed as anti - foaming agents and sludge biomass removers.

The Congo red binding characteristic (Crb+), which is often employed as a measure of absorption and has been connected directly to pathogenicity and was widely used in vitro to investigate the pathogenicity of bacterial isolates [30]. Due to the presence of a close link between Congo red binding and hemin, which serves as an iron supply for the organisms, and protoporphyrin, the main component of the cell surface protein. Because of their structural similarity, they might be employed interchangeably to discriminate virulent and non-virulent isolates in solid media [31].

Certain Gram-negative bacteria isolates that are pathogenic attach the dye Congo red from solid agar media and generate red colonies (Crb+), while isolates that do not absorb the dye generate white colonies (Crb-) and are not pathogenic. In this study, among organisms with virulence associated with the ability to bind Congo red are *E. coli* and *P. aeruginosa*, these results are in agreement with the findings recorded by Mahgoub et al. [32]. The most pathogenic bacteria are *P. aeruginosa* followed by *E. coli* with 96% and 83% respectively. These findings suggested that all of the bacteria recovered were highly pathogenic, posing a significant risk to humans and the environment.

The antibiotic resistance of bacteria isolates was determined by calculating the total number and percentages of resistant bacterium isolates from water samples collected from various locations. Cephalothin resistance was observed for 97% of all bacterial isolates. Clindamycin 94.4%, erythromycin 77%, vancomycin 63%, nitrofurantoin 62%, trimethoprim/sulfamethoxazole 57.2%, amoxycillin/clavulanic acid 54.4%, chloramphenicol 52.8%, kanamycin 47%, and tetracycline 33%. Finally, ofloxacin resistance was the lowest percentage, with only 16% of all bacterial isolates demonstrating resistance.

These findings are consistent with those of Mahgoub et al. [32], who found cephalothin resistance to be (98.2%), followed by clindamycin (95.2%), methicillin (91.7%), erythromycin (79.4%), tobramycin and chloramphenicol (66.2%), sulfa/trimethoprim (63.2%), and vancomycin (62.3%). Multiple antibiotic resistance (MAR) values for detected bacterial species obtained from water samples from various sites revealed that *P. aeruginosa* (0.87) had the highest MAR values, followed by *E. coli* (0.58). Krumperman [20] reported that MAR values greater than 0.25 indicated a substantial danger of pollution. Sadly, all of these estimated MAR scores were clearly over the high-risk level (0.25). According to the MAR index calculated at each sampling location, all the sites included in this investigation were at high hazard at various degrees.

In light of the previously discussed results from antibiotics susceptibility tests and measurement of MAR index values, it was clear that the pollution levels suggested by physicochemical and bacteriological analyses play a significant role in the incidence of antibiotic-resistant bacteria (ARB) and that the spread of these bacteria is dependent on pollution levels.

For identification and genetic level conformation, two bacterial isolates *E. coli* and *P. aeruginosa* were isolated from various locations in this study Bahr El-Baqar Drain and lake Manzala. DNA was isolated and amplified using the traditional PCR method with particular primer sequences for *E. coli* and *P. aeruginosa* 16s rDNA. Several studies have used the 16s rDNA sequence to investigate the genetic diversity of *E. coli* and *P. aeruginosa* isolates [32, 33, 7]. Using the DNAMAN tool, the sequences were matched to bacterial species in the GenBank database and identified as *P. aeruginosa* strain R2 (Accession No. MK071734.1) and *E. coli* strain R1 (Accession No. MK064165.1).

Mainly, phages are isolated from locations that serve as habitats for the host bacteria, such as sewage, soil, and water. According to our findings, the number and behaviour of phages were strongly influenced by the density of their hosts. After enrichment, phages specific for *E. coli* strains and *P. aeruginosa* strains were collected from all studied sites with concentrations ranging from 10^7^ to 10^9^ pfu ml^− 1^ for *E. coli* and 10^6^ to 10^8^ pfu ml^− 1^ for *P. aeruginosa* phages, respectively, and designed as C1 to C3 phages specific for *E. coli* and Ps1 to Ps3 phages specific for *P. aeruginosa*. these findings were similar to those reported by Mahgoub et al. [32], who collected sewage water from wastewater treatment plants (WTs) in Egypt’s El-Sharkia Governorate. On the other hand, the titre of phages for *E. coli* and *P. aeruginosa* that detected by El-Dougdoug et al. [33] which recorded 10 ^2^ pfu ml^− 1^ was so far from these results, these differences due to the variance in density of bacteria specific to phages.

All isolated phages specific for *E.coli* and *P.aeruginosa* form a circular, clear plaque with a diameter from 1 to 5 mm, which is consistent with the findings of El-Dougdoug et al. [33], who found that all isolated phages from wastewater samples which collected from New Cairo, Gabal El-Asfar, and Helwan formed circular plaques with diameters ranging from 1 to 5 mm. C1, C2, and C3 are the names of the *E. coli*-specific phages while *P. aeruginosa*-specific phages were known as Ps1, Ps2, and Ps3. The single plaque isolation procedure was used to obtain a single plaque from these plaques. The bacteriophages were propagated using the liquid propagation method to obtain huge volumes of bacteriophage suspension. Finally, polyethylene glycol sedimentation was used to purify the generated bacteriophage stock lysates, yielding purified, concentrated bacteriophage stock lysate [32, 34]. After chemical purification, the high titer (10^12^ pfu/ml) of *E. coli* and *P. aeruginosa* bacteriophage stock produced was matched with those obtained by El-Dougdoug et al. [33].

The morphological characteristics of isolated phages revealed varied lengths by using TEM. According to the **International Committee on Virus Taxonomy**, all isolated phages belonged to the *Siphoviridae* and *Myoviridae* families. The laters contain a large number of phages that infect species of the Enterobacteriaceae and Pseudomonadaceae groups, which agrees with our findings [32, 33, 7]. The host range of the isolated bacteriophage may be narrowly restricted within a bacterial species. According to the host range, all coliphages were lysosensible to *E. coli* R1 and *E. coli* ATCC 25,922. However, none of the separated phages was able to lyse *E. coli* ATCC 10,536. While all *pseudomonas* phages were able to lyse *P. aeruginosa* strain R2 and *P. aeruginosa* ATCC 27,853. These findings were similar to those obtained by Mahgoub et al. [32].

In this study, most plaques were formed after heating at 40 ^o^C. As the temperature increased, the vitality of phages decreased. Heat stability was found in all three *E. coli* phages. These findings were not matched with results obtained by Samhan et al. [35], who isolated three *E. coli* phages (ECP) from the Ismailia Canal Mostorod section that were heat sensitive. On the other hand, the infectivity of *P. aeruginosa* phages P2 and P3 was disappeared after a 10-minute exposure to 80 °C, while p1 not detected after a 10-minute exposure to 100 °C.

Six isolated phages of *E. coli and P. aeruginosa* were viable at different pH levels ranging from 5 to 10; these data are similar to those obtained by Samhan et al. [35]. The phages lost their viability at low pH levels due to The acid denaturation the of protein which caused the phage inactivation. Bacteriophages have been effectively used in the treatment of wastewater as biological agents or tracers for their specific bacterial hosts [8]. The effects of identified phages on reducing the viable count of *E. coli* and *P. aeruginosa* bacteria in sewage water samples were investigated. Bacterial infection with the examined phages was tracked for 24 h. The decline in bacteria achieved by phages was compared to a control group. With time, phage infection resulted in a significant reduction in *E. coli and P. aeruginosa* production. The removal efficiency (%) for *E. coli and P. aeruginosa* strains increased throughout time and peaked at 24 h after incubation with the phages cocktail. After 24 h of treatment with a mixture of phages, *E. coli and P. aeruginosa* microorganisms in wastewater samples showed a 99.5% reduction.

These results are compatible with Elbahnasawy et al. [7] who studied a new coliphage for biocontrol of MDR *E. coli* and coliforms in wastewater. Total and faecal coliforms were reduced by at least 30-fold in all water samples after 12 h of treatment with a combination of coliphages. Saad et al. [36] used bacteriophage to inactivate pathogenic bacteria from wastewater. The bacterial reduction rate is 5 times higher than that determined for the control filter. These results show the positive impact of the phage on bacterial inactivation and the improvement of water treatment.

Furthermore, numerous authors proposed that bacteriophages be used in conjunction with bacterial markers as a trustworthy sign of faecal contamination. It is now necessary to pay attention to its effectiveness in the control and lysis of harmful bacteria that outperform antibiotics.

## Conclusion

This study discussed the diversity of phages and their potential role in improving water quality by reducing the number of pathogenic multi-drug resistant gram-negative bacteria such as *E. coli* and *p. aeruginosa*. Results demonstrated the high efficacy of lytic phages in controlling the growth of MDR *E. coli* and *p. aeruginosa* in drainage water of the Bahr el baqar drain. Our approach proved to be simple, fast, cost-effective, and specific for the reduction wide range of these strains, being important water-borne pathogens for public health. We recommend the use of advanced molecular techniques for the rapid and accurate identification of phages. In addition, the future production of commercial phage mix to utilize it as bacterial bio-control agents in different water resources. Hence, our study recommends the protection of surface water resources from pollution by enforcement of the actual application of LAW 48∕1982 regarding the protection of the river Nile and waterways from pollution.

## Electronic supplementary material

Below is the link to the electronic supplementary material.

Supplementary Material 1

## Data Availability

The sequence reads of *E. coli* and *p. aeruginosa* strains are available in GenBank under accession numbers **MK064165** and **MK071734** (https://www.ncbi.nlm.nih.gov/nuccore/MK064165.1 and https://www.ncbi.nlm.nih.gov/nuccore/MK071734.1).

## Abbreviations

BBD: Bahr El-Baqr drain
MAR: multiple antibiotic resistances
MDR: multidrug-resistant
MDRGNB: multidrug resistant Gram-negative bacteria
BCM: billion cubic meters
CLEQM: Central Laboratory for Environmental Quality Monitoring
NWRC: National Water Research Center
CRA: Congo Red Agar
TIP: Thermal inactivation point
MSA: multiple sequence alignment
NCCLS/CCLS: National Committee for Clinical Laboratory Standards/ Clinical and Laboratory Standards Institute
ATCC: American Type Culture Collection
EMB: Eosin Methylene Blue
mTEC: membrane Thermotolerant *E. coli*
M-PA-C: modified *P. aeruginosa* culture
