# Supplementary material for: Biocontrol of multi-drug resistant pathogenic bacteria in drainage water by locally isolated bacteriophage
Source: BMC Microbiol. 2023 Apr 26;23:118. doi: 10.1186/s12866-023-02847-4 (PMC10131467; doi:10.1186/s12866-023-02847-4)
Supplement: Supplementary file 1 — Supplementary Material 1 [file 12866_2023_2847_MOESM1_ESM.docx]

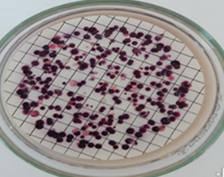

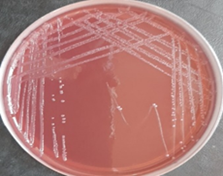

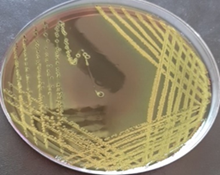


**Supplementary figure 1**: *E. coli* colonies on modified mTEC, MacConkey and EMB agar medium, respectively.


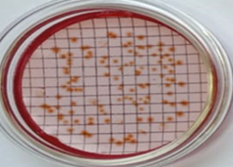

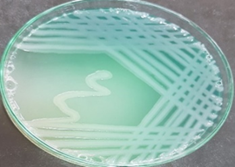


**Supplementary figure 2:** *Pseudomonas aeruginosa* colonies on M-PA-C and cetrimide agar medium, respectively.

**A
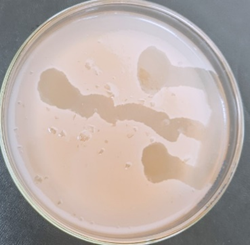
 B
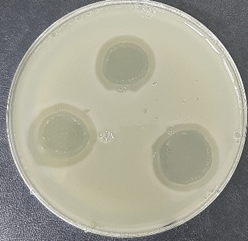
**

**Supplementary figure 3:** Bacterial lysis of *E. coli* (A) and *P. aeruginosa* (B) by their compatible bacteriophages after spot test.

**A**)
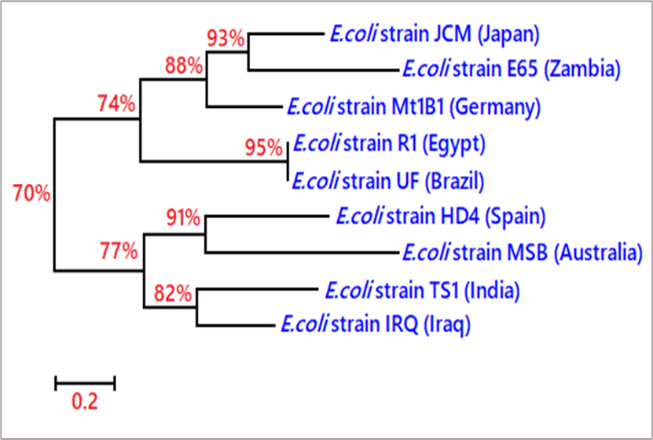
 **B)**
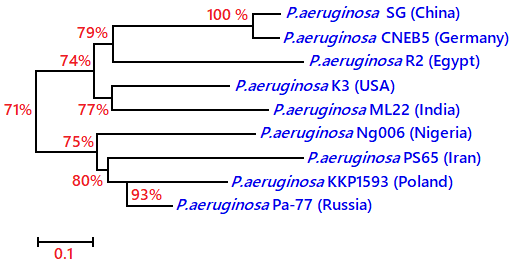


**Supplementary figure 4**: Phylogenetic tree pf identified bacterial strains (A- *E. coli* & B- *P.aeruginosa*) and related strains published in GenBank based on 16S-rDNA sequences.

**Supplementary table 1: Antibiotics used for sensitivity test.**

| **Class of antibiotics** | **Scientific Name** | **Trade Name** | **Symbol** |
| --- | --- | --- | --- |
| Penicillin's | Amoxycillin/Clavulanate (30ug) | Augmentin | AG |
| Cephalosporins | Cephalothin (30ug) | Keflin | KF |
| Glycopeptides | Vancomycin (30ug) | Vancocin | VA |
| Aminoglycosides | Kanamycin (30ug) | Kanatrex | K |
| Tetracyclines | Tetracycline(30ug) | Tetracycline | TE |
| Macrolides | Erythromycin(10Ug) | Erythromycin | E |
| Lincosamides | Clindamycin(30ug) | Lincocin | DA |
| Quinolones | Ofloxacin(10Ug) | Tarivid | OFX |
| Sulfa drugs | Trimethoprim/Sulfameth-oxazole (25Ug) | Septrin | SXT |
| Nitrofurans | Nitrofurantoin (30ug) | Colifuran | F |
| Chloramphenicol | Chloramphenicol (30ug) | Chloramphenicol | C |

**Supplementary table 2: Multiple antibiotic resistance (MAR) index for bacteria isolated from collected sewage water samples.**

| **Bacterial isolate** | **MAR index** |
| --- | --- |
| ***E. coli*** | **0.58** |
| ***Pseudomonas aeruginosa*** | **0.87** |
